# Bioactive Glasses with Low Ca/P Ratio and Enhanced Bioactivity

**DOI:** 10.3390/ma9040226

**Published:** 2016-03-24

**Authors:** Marco Araújo, Marta Miola, Giovanni Baldi, Javier Perez, Enrica Verné

**Affiliations:** 1 Colorobbia España S.A, Carretera CV-160, Vilafamés 12192, Spain; marco.filipee233@gmail.com (M.A.); jperez@colorobbia.es (J.P.); 2 Ce.Ri.Col, Centro Richerche Colorobbia, Via Pietramarina 123, Sovigliana (FI) 50053, Italy; baldig@colorobbia.it; 3 Politecnico di Torino, Applied Science and Technology Department, Corso Duca degli Abruzzi 24, Torino 10129, Italy; enrica.verne@polito.it; 4 Present affiliation: Università del Piemonte Orientale, Department of Health Sciences, Via Solaroli 17, Novara 28100, Italy

**Keywords:** bioactive glasses, bioactivity, Ca/P ratio, densification, sintering

## Abstract

Three new silica-based glass formulations with low molar Ca/P ratio (2–3) have been synthesized. The thermal properties, the crystalline phases induced by thermal treatments and the sintering ability of each glass formulation have been investigated by simultaneous differential scanning calorimetry-thermogravimetric analysis (DSC-TG), X-ray diffraction (XRD) and hot stage microscopy (HSM). The glasses exhibited a good sintering behavior, with two samples achieving shrinkage of 85%–95% prior to crystallization. The bioactivity of the glasses in simulated body fluid (SBF) has been investigated by performing XRD and Fourier transform infrared spectroscopy (FTIR) on the samples prior and after immersion. The glasses with lower MgO contents were able to form a fully crystallized apatite layer after three days of immersion in simulated body fluid (SBF), while for the glass exhibiting a higher MgO content in its composition, the crystallization of the Ca–P layer was achieved after seven days. The conjugation of these properties opens new insights on the synthesis of highly bioactive and mechanically strong prosthetic materials.

## 1. Introduction

Tissue engineering has emerged as a promising approach for repair and regeneration of tissues and organs, lost or damaged as result of trauma, injury, disease or aging [1]. For many years, autografts, allografts and xenografts have been used as prosthetic materials in the field of tissue engineering [2]. However, most of these implants suffer from problems of interfacial stability with host tissues, biomechanical mismatch of elastic moduli, production of wear debris and maintenance of blood pressure, immune rejection or viral transmission from the donor [2,3]. To overcome many of these problems, in the last few years, synthetic grafts as hydroxyapatite, tricalcium phosphate and bioactive glass and glass-ceramics have been highly investigated, focusing attention on their ability to bond to the surrounding osseous tissue and to enhance bone formation [4]. In the case of bioactive glasses, it has already been demonstrated that the mechanism of bonding to living tissues involves 11 reaction steps [5]. In this cascade of reactions, the first five steps occur essentially on the glass surface and involve rapid ion exchange of Na^+^ with H_3_O^+^, followed by the formation of surface silanols, that undergo polycondensation creating a silica gel layer which, in turn, provide a high number of active sites for heterogeneous nucleation and crystallization of a biologically reactive hydroxy-carbonate apatite layer. Due to its high similarity to the inorganic mineral phase of bone, this layer provides an ideal environment for colonization of osteoblasts, proliferation and differentiation of the cells to form new bone, creating a mechanically strong bond with the implant surface [5]. According to Hench, glasses having <60 mol % SiO_2_ in their composition, high CaO and Na_2_O contents and high Ca/P ratio are bioactive [6], whereas glasses with much lower Ca/P ratio would not bind to bone [7]. These rules were well expressed on the formulation of the one of the most popular bioactive glasses, the 45S5 (Bioglass^®^), whose composition (wt %) is 45% SiO_2_, 24.5% CaO, 6% P_2_O_5_ and 24.5% Na_2_O, having a molar Ca/P ratio of 5. Other Bioglass^®^-based compositions, having a fixed amount of P_2_O_5_ were developed, including the 52S4.6 and 55S4.3 [8], KGS Ceravital [9], S45P7 [10] and, more recently, the glass S53P4 [11]. Although all these glasses exhibited a good bioactivity, and some of them reached commercial success, they have some limitations in terms of their physico-chemical properties, mainly due to their deficient thermal behavior that provides a negative impact on their sintering ability and mechanical properties, limiting the application of these glasses as powder and granule bone fillers rather than 3D load bearing implants [12,13]. In this context, a series of glasses in the system CaO-MgO-SiO_2_-Na_2_O-P_2_O_5_-CaF_2_ exhibiting good sintering properties in a temperature range below crystallization were developed [14,15]. Other glasses in this system exhibiting good bioactivity include the AP40 [16], 13-93 [17] and CEL2 [18]. A common feature of all these glasses is the amount of P_2_O_5_, which covers a concentration range between 4 and 11 wt % and a Ca/P > 4. Very little work has been done on designing formulations having high amounts of P_2_O_5_, or lower Ca/P ratio. One of the few studies performed in this field revealed that glasses in the system SiO_2_-CaO-P_2_O_5_-Na_2_O containing 10 wt % P_2_O_5_ exhibited good mechanical properties (bending strength between 120 and 190 MPa and compressive strength 600–900 MPa), which is considerably higher than the one observed for 45S5 glass-ceramics compacts (bending strength 30 MPa and compressive strength 37 MPa) [19]. Besides their biocompatibility, these low Ca/P glasses were able to form a tight chemical bond with bone [20,21,22]. In terms of bioactivity, it is generally well established that glasses having a high amount of P_2_O_5_ in their composition exhibit a high polymerization of the silicate network, which in turn has a negative impact on glass bioactivity [23,24,25]. However, one of the few studies on the influence of P_2_O_5_ content on the bioactivity of glasses in the system SiO_2_-CaO-P_2_O_5_-Na_2_O suggests that although the glass network connectivity increases, an increase in the rate of formation of the apatite layer may be also observed in glasses with a high amount of P_2_O_5_ (~10 wt %) [26]. In this study, glasses with 6.8 wt % P_2_O_5_ content were found to form a crystallized apatite layer after 16 h of immersion in SBF solution, exhibiting intense, narrow and split peaks on the zone of 550 cm^−1^, detected by FTIR, whereas for 45S5 bioglass, the phosphate peak could hardly be resolved from the background after 1 day immersion. Some studies regarding the SiO_2_ and MgO content of bioactive glasses were also published [27,28]. While Oliveira *et al.* [27] studied the role of MgO on the surface reactivity of glasses belonging to the SiO_2_-P_2_O_5_-CaO-MgO system, reporting an increasing surface activity with increasing MgO/CaO ratio, Verné *et al.* [28] have prepared and characterized a set of glasses and glass-ceramics belonging to the SiO_2_-P_2_O_5_-CaO-MgO-K_2_O-Na_2_O system, reporting very low influence of the MgO/CaO ratio on the bioactivity mechanism. These observations open a new way towards the rational design of glasses with high bioactivity and guides to a new interpretation of their composition-structure relationship. In recent years, the inclusion of CaF_2_ in bioactive glass formulations has also attracted great interest due to the possibility of formation of a fluoroapatite crystalline phase [29]. Fluoroapatite has a higher chemical stability than hydroxyapatite and is determinant in the prevention of caries [30]. Furthermore, fluorine is well known to prevent dental decay by inhibiting enamel and dentine demineralization, enhancement of remineralization and inhibition of bacterial enzymes [31].

Herein, we present the design and development of three new bioactive glass formulations in the system SiO_2_-CaO-P_2_O_5_-Na_2_O-K_2_O-MgO-CaF_2_ with low CaO/P_2_O_5_ ratio, and different MgO/CaO ratio. These new formulations belong to the system of the apatite-wollastonite glass-ceramics, which have high potential as mechanically strong prosthetic implants [27,28]. The thermal behavior and bioactivity of the newly developed glasses as well as the effect of MgO/CaO ratio on these parameters were investigated.

## 2. Results and Discussion

Three new glass formulations in the system SiO_2_-P_2_O_5_-CaO-Na_2_O-MgO-CaF_2_ having a low molar Ca/P ratio (2.3–2.8) were developed and their physico-chemical properties investigated, regarding their possible application as prosthetic materials.

As represented in Table 1, a common characteristic between these new glass formulations is related with their high P_2_O_5_ amount (14–17 wt %), which highly influences the physico-chemical properties of the material [20,21]. Taking into account this feature, the thermal properties of the newly developed glasses were investigated using DSC-TG and HSM. As glass B32 was developed from glass B31 by substitution of CaO with MgO, the resultant DSC and HSM curves were combined in the same graph to allow a better analysis on the influence of this substitution in the thermal behavior of the glasses.

**Figure 1:**
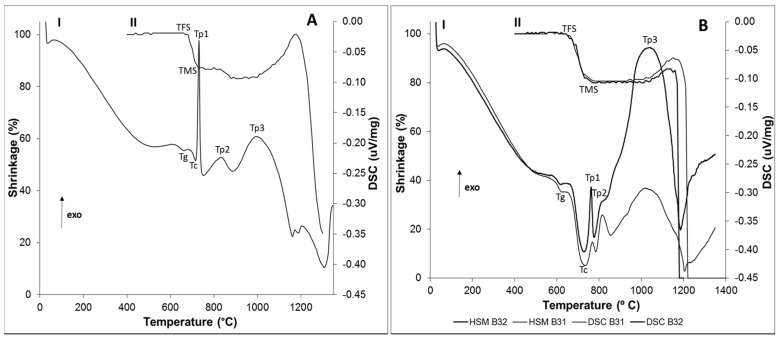
DSC (I) and HSM (II) curves of B4 (**A**), B31 and B32 (**B**). *T_g_*: Temperature glass transition; *Tc*: onset of crystallization; *Tp1*–*Tp3*: Maximum temperatures of crystallization; *TFS*: Temperature first shrinkage; *TMS*: Temperature maximum shrinkage.

Figure 1B shows that the newly developed glasses B31 and B32 exhibit a single-stage shrinkage and a good sintering behavior, this last one supported by their high densification degree prior to the onset of crystallization. The data provided by Figure 1 are summarized in Table 2.

**Figure 2:**
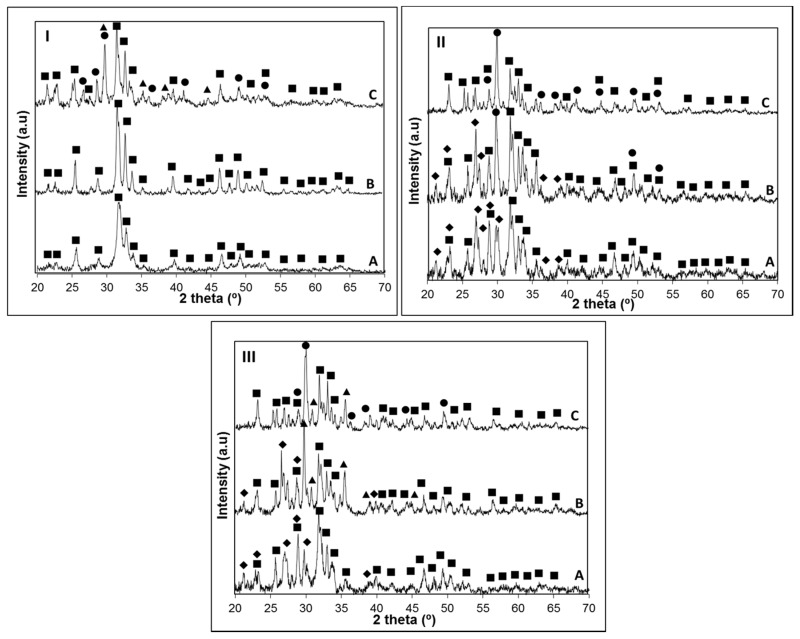
(**I**) Bioglass B4 crystallized at: A, 731 °C; B, 832 °C; and C, 993 °C for 1 h (β = 5 °C·min^−1^). (**II**,**III**) Bioglass B31 (**II**) and B32 (**III**) crystallized at: A, 765 °C; B, 814 °C; and C, 1020 °C for 1 h (β = 5 °C·min^−1^). XRD: Apatite (■), Diopside (▲), Wollastonite (●), and Devitrite (♦).

According to HSM analysis of glasses B31 and B32 (Figure 1B, curve II), 86% and 93%, respectively, of the total densification (19.3% and 19.5%) exhibited by these glasses was achieved below the *Tc*. Such behavior is nowadays considered an essential characteristic for the production of viscous flow sintered scaffolds with high mechanical properties [15]. The glass B4 exhibited the worst sintering behavior, presenting a two-stage densification and shrinkage of 9.25% (out of 13.7%) around the onset of crystallization (*Tc*). This lower sintering ability (*Sc* = −15), represented as the difference between the temperature of onset of crystallization and temperature of maximum shrinkage, may be related with the higher amount of P_2_O_5_ present in this glass formulation, which often guides to an increase on the extent of polymerization of the silicate glass network, resulting in the reinforcement of the glass structure [6]. Consequently, the temperature required to promote the densification of the glass is usually higher, guiding to a natural decay in glass shrinkage and, consequently, in its sintering performance. The characteristic viscosity points of the synthesized glasses were determined by DSC. By analyzing Figure 1A curve I and Figure 1B curve I, it can be observed that *T_g_* was higher for glass B4 (662 °C), containing the highest amount of P_2_O_5_, in comparison with glasses B31 (620 °C) and B32 (616 °C). Although this could arise from a possible higher network connectivity of glass B4, it cannot be ruled out that it is due to the lower wt % of CaF_2_ in the glass formulation. It has been already studied that CaF^+^ species form weak electrostatic forces between the non-bridging oxygens of the glass network (similar to the ones of the fluxing agents), decreasing the bonding strength between the silicate ions in the network and consequently, the temperature required for the glass to lose its cohesiveness [32,33]. Furthermore, the high similarity between the ionic radius of fluorine (1.36 Å) and the one of the oxygen (1.40 Å) may guide to the replacement of bridging oxygens with non-bridging fluorides, resulting in the disruption of the glass network [30,34]. As a result, glass B31 and B32, both having a higher amount of CaF_2_, would benefit of a lower *T_g_*, as demonstrated in Figure 1B. The obtained DSC data (Figure 1A curve I and 1B curve I) show that all the glasses present a similar crystallization behavior, exhibiting three main crystallization peaks. However, considering that the presence of CaF^+^ species softens the glass network, it would not be expected that glasses B31 and B32 have higher Tp1 temperatures than glass B4. To discuss this behavior, another subject has to be brought into account, which is the increase in the Ca/P ratio of the glass as CaF_2_ increases. It is already known that fluorapatite undergoes phase separation below crystallization [35]. Thus, glasses with higher amount of P_2_O_5_ (as glass B4) would present a lower activation energy for the nucleation of fluorapatite, as the addition of P_2_O_5_ induces phase separation in the glass network. This phenomenon facilitates the growth of fluorapatite crystals due to an enriched composition of the phase in the components of this mineral, specifically P^5+^ and Ca^2+^, guiding to a lower *Tc* and *Tp1*. On the other side, the higher amount of CaF_2_ present in the composition of glasses B31 and B32 induces the formation of a higher number of nuclei per volume of glass, in comparison with sample B4. Consequently, the network connectivity of the glass surrounding the nucleus will be higher, creating a barrier to crystal growth and thus, guiding to an increase in the activation energy required for the crystallization of fluorapatite [36]. Thus, it is reasonable that a lower *Tp1* is observed in the glass with the higher P_2_O_5_ content and lower CaF_2_ composition. The crystalline phases nucleated at *Tp1*, *Tp2* and *Tp3* on the three glasses were investigated by XRD (Figure 2).

The results obtained in Figure 2 are summarized in Table 3 for a better comparison of the crystalline phases found on the three glasses.

XRD analysis of the crystallized samples allowed us to conclude that the apatite phase Ca_5_(PO_4_)_3_(OH, F) was present in all the glass-ceramics (PCPDF 98-007-7966). While on the glass with the highest P_2_O_5_ content this was the only crystalline phase present at *Tp1*, in B31 and B32 glasses devitrite (Na_2_Ca_3_Si_6_O_16_; PCPDF 98-007-7397) crystals grew along with the apatite phase. As observed in Table 3, there is a clear trend for the precipitation of calcium-rich crystalline phases, such as diopside (MgCaSi_2_O_6_; PCPDF 98-002-1772), wollastonite (CaSiO_3_; PCPDF 98-002-1772) or devitrite (Na_2_Ca_3_Si_6_O_16_; PCPDF 98-004-6279) as the content in P_2_O_5_ decreases. This was rather expected, since the higher amount of non-bonding oxygens in the glasses B31 and B32, arising from a potentially lower network polymerization, favors the formation of crystalline phases containing modifiers (CaO, CaF_2_, MgO) and/or fluxing agents (Na_2_O, K_2_O). These calcium-silicate phases were detected in glass B4 at temperatures around 1000 °C, due to a higher resistance of the glass network to deformation, which is also in accordance with the HSM results (Figure 1II). In glass B32 at *Tp2*, the effect of replacing CaO by MgO favored the formation of a magnesium-calcium silicate crystalline phase (diopside) instead of wollastonite (B31). The obtained data suggest that by adjusting the Ca/P ratio, a control over the crystalline phase composition and bioactivity can be achieved. In order to get conclusions on the influence of the glass composition and Ca/P ratio in its bioactivity, without having the interference of such crystalline phases, the apatite forming ability of the glasses was investigated using amorphous powdered glass compacts, sintered at a temperature below crystallization. The good sintering behavior of the glasses allowed the preparation, at low temperatures, of sintered glass pellets that could be easily manipulated.

**Figure 3:**
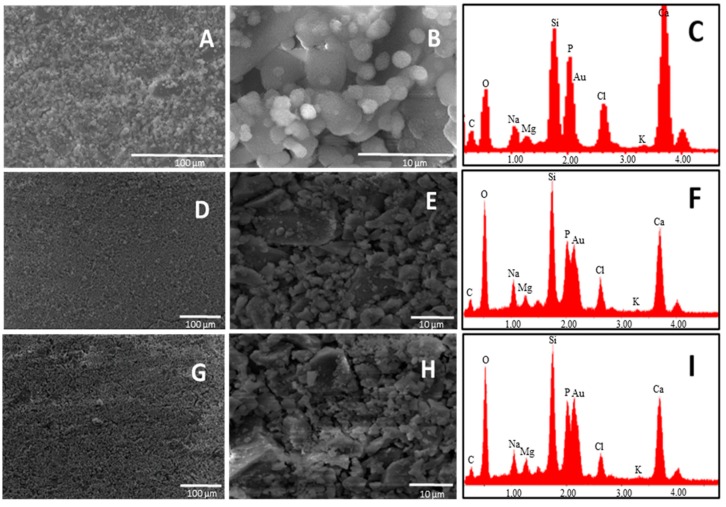
SEM-EDS on glasses: B4 (**A**–**C**); B31 (**D**–**F**); and B32 (**G**–**I**) after one day of soaking in SBF. Magnification is 500× (left) and 5000× (middle). EDS analysis was done on the whole area of images at 500×. Samples were coated with Au.

At this stage, it is useful to remember the mechanism of formation of the hydroxyapatite-like layer on the surface of the bioactive glasses, which firstly occurs by release of Ca^2+^, Mg^2+^, and Na^+^ ions into SBF solution via an exchange with H^+^ ions present in the fluid (stage 1), followed by the formation of surface silanols, which in turn undergo polycondensation resulting in the formation of silica gel layer on the glass surface (stage 2). This layer immediately combine with Ca^2+^ ions in the fluid to form an amorphous calcium silicate layer (stage 3), which reacts with phosphate ions present in SBF guiding to the formation of an amorphous calcium-phosphate layer with low Ca/P ratio (stage 4). This phase later transforms into bone-like apatite crystal (stage 5), increasing its Ca/P ratio and incorporating minor ions such as Na^+^, Mg^2+^ and Cl^−^ [1,37]. SEM analysis performed on the glass surface after one day of soaking in SBF revealed that the glass having the lowest Ca/P ratio exhibited a higher appetence for precipitation of calcium phosphate crystals (Figure 3).

**Figure 4:**
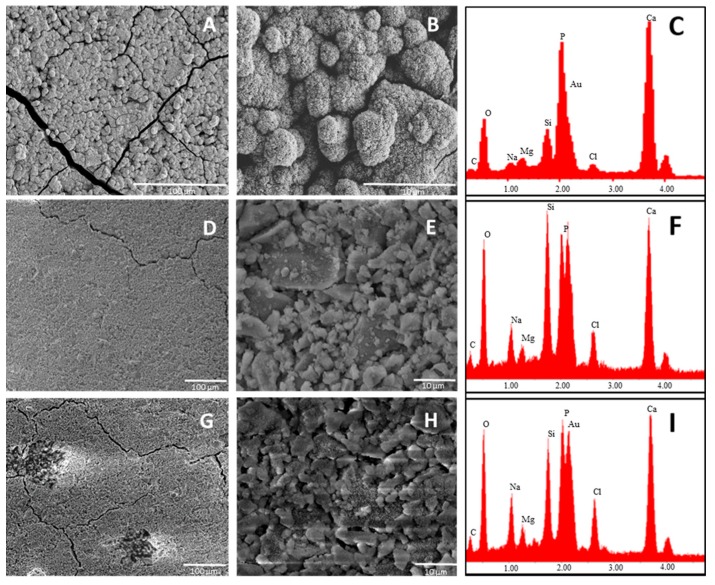
SEM-EDS of glasses: B4 (**A**–**C**); B31 (**D**–**F**); and B32 (**G**–**I**) after three days of soaking in SBF. Magnification is 500× (left) and 5000× (middle). EDS analysis was done on the whole area of images at 500×. Samples were coated with Au.

SEM pictures of B4 at 5000× magnification showed the presence of calcium phosphate crystals covering all the surface of the glass just after one day of soaking in SBF. By analyzing the surface of the other two glasses (B31 and B32), it seems that these kind of crystals are also present on them, but in lower amount. This was confirmed by EDS analysis where the lowest Si/P and Si/Ca ratio was detected for the surface layer on B4. Such results suggest that a Ca–P layer with higher thickness was able to form on the surface of B4, highlighting its higher apatite forming ability when compared with glasses B31 and B32. This behavior is in contrast with the theory established by Hench on the design of bioactive glasses, which suggested that glasses having a Ca/P ratio < 5, as the popular 45S5, would not be able to form a tight bond to bone through the formation of an apatite layer [7]. A similar trend in bioactivity was maintained after three days soaking in SBF solution (Figure 4).

The higher amount of surface cracks (after drying the sample at 100 °C) observed on sample B4, characteristic of the growth of a silica gel layer on the first stages of bioactivity, is also evidence of the higher bioactivity of this sample. Comparing glasses B31 and B32, the apparently lower Si/P observed in the last suggests the presence of a calcium phosphate layer with higher thickness after three days of soaking in SBF solution, which could be linked to a higher bioactivity.

**Figure 5:**
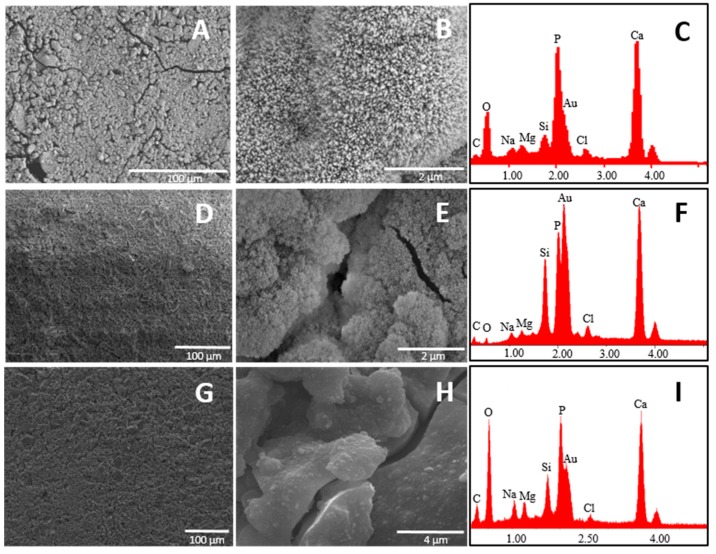
SEM-EDS of glasses B4 (**A**–**C**); B31 (**D**–**F**) and B32 (**G**–**I**) after seven days of soaking in SBF. Magnifications are 500× (left) and 20,000× (middle). EDS analysis was done on the whole area of images at 500×. Samples were coated with Au.

SEM-EDS results revealed that after seven-day immersion in SBF, the surface layer of glass B4 still exhibits a Ca:P 1:1, while the one present in glass B32 shows a Ca/P > 1, suggesting the formation of the calcium deficient apatite on the first (Figure 5). The higher P_2_O_5_ content of B4 explains this behavior.

**Figure 6:**
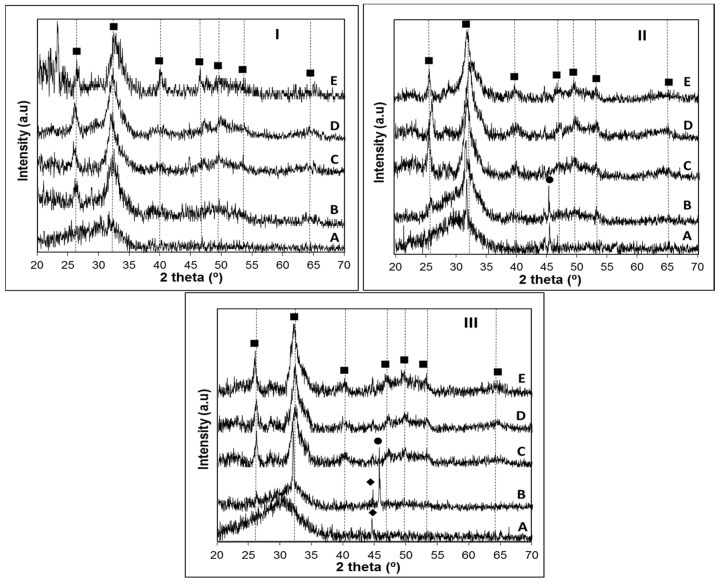
XRD analysis of glass: B4 (**I**); B31 (**II**); and B32 (**III**) after 1 (A), 3 (B), 7 (C), 14 (D) and 21 (E) days bioactivity. XRD: Apatite (■), NaCl (●), CrFeNi support (♦).

As Ca^2+^ interacts preferentially with the non-bonding oxygens from phosphates rather than with silicates, a higher quantity of phosphate units in the glass structure would decrease the migration kinetics of Ca^2+^ to the glass surface, favoring the formation of a layer with a deficient composition in calcium. At this stage, however, due to the low Si/P ratio observed, all the glasses were already covered by a considerable amount of Ca-P layer. The phase composition of the surface layer on the glasses was also followed by XRD in order to obtain information about the identity of the layer and on its amorphous/crystalline state (Figure 6).

**Figure 7:**
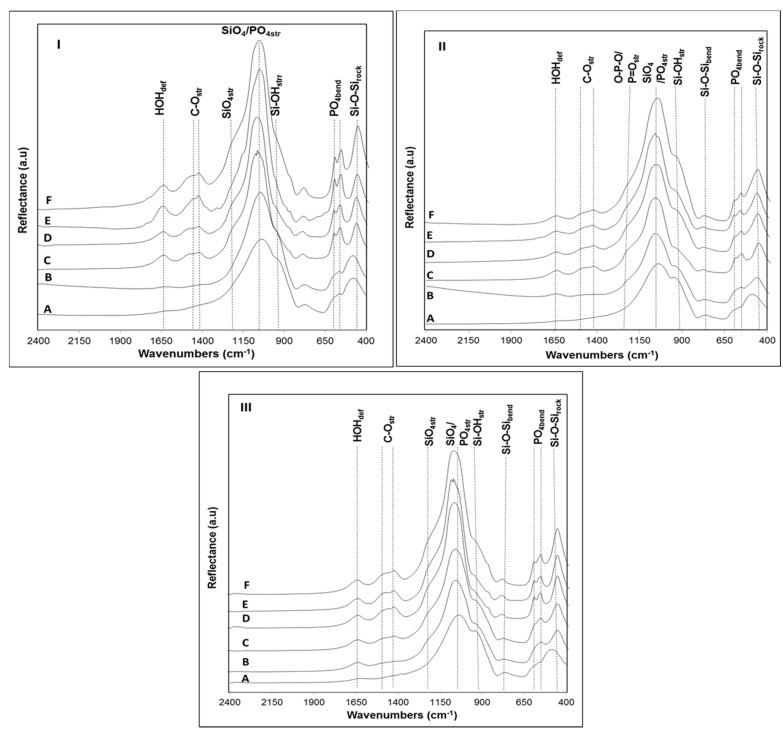
FTIR analysis of glass: B4 (**I**); B31 (**II**); and B32 (**III**) before (A) and after 1 (B), 3 (C), 7 (D), 14 (E) and 21 (F) days bioactivity.

The obtained XRD patterns (Figure 6) confirmed the formation of a crystalline layer on the surface of all the investigated glasses after a maximum of seven days immersion in SBF, which could be identified as an apatite-like layer (PCPDF 98-007-7966). In glass B4, the position of the broad band near 2θ = 32°, where the most intense peak of hydroxyapatite usually appears, may suggest that this layer could be partially crystallized just after one day of soaking in SBF solution (Figure 6IA). However, a clearly crystalline state of this layer was observed after immersion of the B4 for three days, where an additional peak appears around 2θ = 25°, together with a broad signal between 2θ = 45°–55° (Figure 6IB). From 3 to 21 days of immersion in SBF solution, it was observed an increase in the crystallinity of the apatite layer, supported by the presence of characteristic hydroxyapatite peaks in the range 2θ = 45° to 55°. Although a similar behavior could be observed for glass B31, in the sample B32 the crystallization of this layer was only observed on samples soaked for seven days in SBF solution, suggesting that the substitution of CaO by MgO delays the crystallization of the Ca-P layer. Additional peaks around 2θ = 32 and 45° observed on samples B31-1D (Figure 6IIA) and B32-3D (Figure 7IIIB) indicate the presence of NaCl (PCPDF 98-002-2556) arising from the SBF. Characteristic peaks of NaCl are also present in sample B31-3D (Figure 6IIB), but the shift of this peak to higher 2θ values, together with its broader appearance suggest that a crystalline apatite layer is also present, which is confirmed by the peak observed at 2θ = 25° in this same sample. In an attempt to obtain additional information on the rate of formation of the Ca-P layer, the surface of samples B4, B31 and B32 after soaking in SBF solution were analyzed by FTIR (Figure 7).

Focusing on the zone corresponding to lower wavelengths (400–610 cm^−1^) on native glass samples (Figure 7A), it was possible to identify peaks associated with Si-O-Si rocking and PO_4_ bending vibrations [38]. While in sample B4 these peaks appear at 496 and 574 cm^−1^, respectively, in the remaining glasses, they are positioned at higher wavelengths, 504 and 602 cm^−1^ (B31) and 505–606 cm^−1^ (B32). Such red-shifting of the PO_4_ bending vibrations in glass B4 may be related to a depolymerization of the phase-separated phosphate network [39]. It has already been reported that upon addition of P_2_O_5_, the P coordination in the phosphate network may be dominated by Q^0^ or Q^1^ units [40]. The high frequency bands (>800 cm^−1^), corresponding to silicon-oxygen stretching vibrations of silicate tetrahedron units, may also provide an idea on the degree of polymerization of the silicate glass network [14]. In the three glasses, the presence of intense peaks around 940 cm^−1^ and 1030 cm^−1^, characteristic of Si–O^−^ stretching vibrations, suggest that the silicate glass structure is dominated by the presence of Q^3^ and Q^2^ tetrahedral units [24,25]. Additionally, the presence of a peak at 738 cm^−1^ confirms the predominance of these units [41]. It is well established that the highest bioactivity from a phosphosilicate glass can be expected if Q*^n^* (Si) units, where n is the number of bonding oxygens (BO), are dominated by chains of Q^2^ metasilicates which are occasionally crosslinked through Q^3^ units, whereas Q^1^ units terminate the chains [24]. Thus, the relatively disrupted silicate network structure of these glasses is also on the basis of their high bioactivity. The presence of non-bonding oxygens in glass structure facilitates the leaching of Ca^2+^ and Na^+^ ions, favoring the formation of a bioactive layer on the glass surface.

After soaking in SBF for one day, some changes in the surface of the glasses have been observed due to the appearance of new peaks in the range 1400–1500 cm^−1^, suggesting the precipitation of carbonated species from the SBF [42]. The changes in the FTIR spectra were accentuated in samples B4 and B31 after soaking for three days immersion in SBF, where a split of the peak at 572 cm^−1^ into two peaks positioned at 574 and 608 cm^−1^ could be observed (Figure 7IC,IIC). This result is usually attributed to the formation of a crystallized apatite layer on the glass surface, indicating that the last stage of bioactivity (stage 5) has been achieved [41]. The absence of these peaks after soaking sample B32 for a similar period of time confirms the lower apatite forming ability of this glass, which is in accordance with the obtained XRD results (Figure 6IIIB). The lower ratio between the peaks at 1055 cm^−1^ (PO_4_^3−^ asym stretching) and 940 cm^−1^ (Si–OH stretching) on glass B32 in comparison with glass B31 during all the bioactivity experiment also supports the lower apatite forming ability of the later, as previously confirmed by SEM-EDS (Figure 4). In all glass samples, the blue-shift of the most intense peak at ~1030 cm^−1^ to 1045–1055 cm^−1^ after one day soaking in SBF solution is characteristic of both P–O stretching vibrations in hydroxyapatite in combination with Si–O vibrations of the silica phase, confirming the fast precipitation of Ca–P species onto the glasses surface [43]. Due to the presence of CO_3_^2−^ species in its composition, the resultant Ca–P layer can be identified as a hydroxycarbonateapatite (HCA) layer [44,45]. The delay in the crystallization of this layer observed for the glass with higher MgO content (B32) may be related to several issues. It is well known that both, MgO and CaO, are considered as glass modifiers, contributing to the modification of the glass properties by increasing the number of non-bridging oxygens and inducing the formation of Si–O–Mg–O–Si or Si–O–Ca–O–Si bonds [36]. Some authors have suggested that the chemical durability of a glass is closely related with the thermodynamic stability of their component oxides [46]. According to literature, the higher Gibbs free energy calculated for the ion exchange reaction between water and a glass of binary system MgO-SiO_2_ (*ΔG*°= −13.888 cal·mol^−1^) in comparison with the analogue determined for a CaO-SiO_2_ system (*ΔG*° = −16.116 cal·mol^−1^) may explain the delay in the leaching of Mg^2+^ to the solution, as well as in the first step of bioactivity mechanism, resulting in a slower transformation of the calcium phosphate layer in a more stable apatite phase [47]. Another theory that could explain this result is related with the ionic field strength *I* (*I* = Z/r^2^*)* of Mg^2+^ and Ca^2+^, which is related with cation charge (Z) and ionic radius (r) [48]. Thus, in this case, the higher ionic field strength of Mg^2+^ (4.73 Å^−2^) in respect to the one of Ca^2+^ (2.04 Å^−2^) suggest that the former would contribute to the formation of tighter glass networks than the later, due to its lower glass modifier character. Furthermore, it was already studied that, apart from being a network modifier, MgO may be also considered as an intermediate oxide that is stored in the network in the form of MgO_4_. The intercalation of this MgO_4_ species in the glass network contributes to an increase in the polymerization of the glass, which in turn results in a decrease of bioactivity [35].

**Figure 8:**
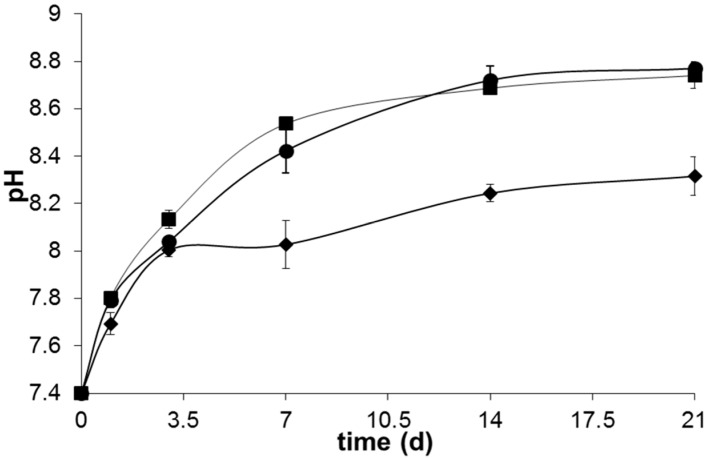
pH changes in SBF after 1, 3, 7, 14 and 21 days of immersion of glasses B4 (♦), B31 (■) and B32 (●).

Although a slower crystallization kinetics of the apatite layer has been observed in glass B32, Figure 8 suggests that the total reconstruction of the glass was totally completed after 14 days of immersion in SBF, where a plateau around pH ~8.2–8.5 was achieved in all the samples.

**Figure 9:**
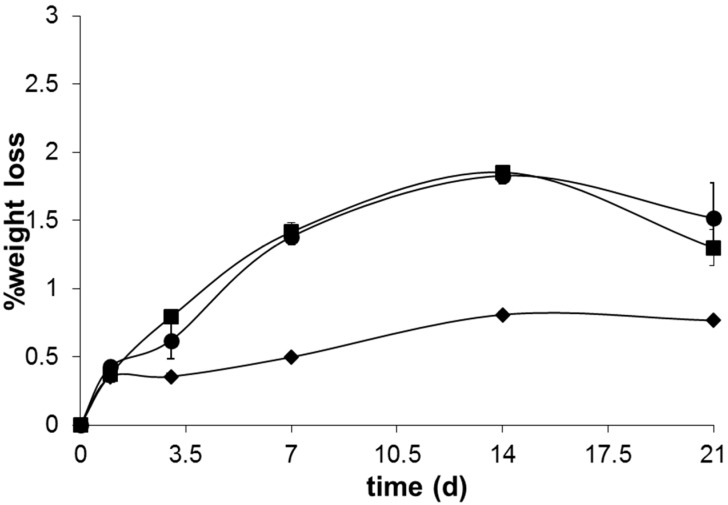
Weight loss of glasses B4 (♦), B31 (■) and B32 (●) after 1, 3, 7, 14 and 21 days of immersion in SBF.

The lower pH exhibited by sample B4 along all the bioactivity experiment may arise not only from the higher amount of P_2_O_5_ in its glass composition, but also from the lower contents of network modifier like Na_2_O and K_2_O. The higher release of phosphate species from the glasses with higher P_2_O_5_ contents probably buffers the alkalinity resultant from the Ca^2+^ and Na^+^ release into the SBF solution maintaining it in a lower pH [26]. Additionally, the increased propensity of Ca^2+^ to coordinate the phosphate network of the glass may also contribute for this behavior. On the other hand, the higher amount of Na_2_O content of B31 and B32 leads to a weaker glass network, increases the fraction of non-bonding oxygens and thus allows an easier ionic exchange between glass and SBF. This results in a higher ionic exchange between the glass and SBF solution in the first stages of bioactivity and a consequent increase in the pH of the medium. The higher amount of MgO present in glass B32 did not have a high influence on the pH changes observed during the bioactivity experiment, apart from a slightly lower pH observed between 3 and 14 days of immersion in SBF solution. This behavior may arise from a decreased precipitation of acidic species (HCO^3−^ and HPO_4_^2−^) from the SBF solution, as a consequence of the lower apatite crystallization kinetics exhibited by this sample. The lower pH registered for sample B4 is also translated in a lower weight loss, as observed in Figure 9.

Although it is known that addition of P_2_O_5_ negatively affects the glass durability due to the facile hydrolysis of P=O bond, this may be counterbalanced by the formation of crosslinked Si–O–P bonds, which could promote glass stability [49,50]. The formation of these crosslinked bonds are usually favored in the presence of higher P_2_O_5_ contents, which is in good agreement with the chemical composition of glass B4 and its consequently lower glass solubility. The presence of MgO in the glass B32 caused a slightly lower solubility of the same until seven days of immersion in SBF solution. This lower glass degradability may arise not only from the higher energy of Mg–O bond, limiting its reactivity and ion leaching, but also from the role of this oxide in glass network, that may to act either as a glass modifier or as an intermediate oxide [35,51]. After 14 days immersion in SBF solution, this glass presents a slightly higher solubility that may be linked with an increased kinetics of ionic exchange between the glass and SBF solution at this stage, which is in accordance with the pH changes observed for this same time period (Figure 9).

The mechanism by which the low Ca/P ratio in glass composition increase their bioactivity may also involve the formation of Ca_3_(PO_4_)_2_ complexes in the glass network on the proximities of the the silica gel layer and facilitate the migration of Ca^2+^ and PO_4_^3−^ species from solution, inducing the crystallization of a hydroxycarbonateapatite layer and a consequent increase in the glass bioactivity [42].

## 3. Experimental Section

Technical grade powders of SiO_2_, Ca_3_(PO_4_)_2_, Na_2_CO_3_, KNO_3_, CaCO_3_, MgO, and CaF_2_ were mixed in the required proportions to obtain glasses with nominal composition (wt %) 40.4% SiO_2_, 7% Na_2_O, 0.3% K_2_O, 30% CaO, 17% P_2_O_5_, 4% MgO, 1.3% CaF_2_ (B4); 40% SiO_2_, 10.5% Na_2_O, 0.5% K_2_O, 30% CaO, 14% P_2_O_5_, 3.0% MgO, 2.0% CaF_2_ (B31); 40% SiO_2_, 10.5% Na_2_O, 0.5% K_2_O, 28.0% CaO, 14% P_2_O_5_, 5.0% MgO, and 2.0% CaF_2_ (B32). The glasses were synthesized by melting the reactants in a platinum crucible at 1400 °C for 2 h (Nannetti electric furnace, Nannetti S.r.l., Faenza, Italy), following a quenching of the melt in water and obtaining a frit. The glass-frit was subsequently wet milled in a high speed porcelain mill using water and Al_2_O_3_ spheres, dried at 100 °C and passed through a sieve to obtain fine glass powders with dimension <64 µm.

In order to detect the characteristic temperatures of the glasses, differential scanning calorimetry (DSC) was measured in a Netzsch STA 449C, in air atmosphere, using Pt-Rh crucibles. For this experiment, 50 mg of the glass powders were heated from room temperature to 1350 °C (β = 3 °C·min^−1^). The parameter β corresponds to the heating rate in °C·min^−1^.

The sintering behavior of the glasses was studied with cylindrical powder compacts with 3 mm height and 1 mm diameter prepared by cold pressing. The samples, placed on an alumina support, were subsequently heated in a hot stage microscope (side-view HSM Misura equipped with an image analysis system and an electrical furnace 1750/15 Leica, Expert System Solutions S.r.l., Modena, Italy) from room temperature to 1350 °C (β = 10 °C·min^−1^) and the size and shape variations were recorded by analyzing images taken in 10 °C intervals between 400 to 1350 °C. The temperature was measured with a Pt/Rh (6/30) thermocouple in contact with the alumina support.

To investigate the type of crystalline phases that grow during heating in the glass matrices, cylindrical green compacts of glass powders with diameter of 1 cm and height of 0.5 cm, prepared by cold uniaxial pressing 750 mg of the glass powders (<64 µm) at 9 bars for 30 s, have been heated up to the crystallization temperatures detected by DSC analysis (β = 5 °C·min^−1^) and maintained at temperature for 1 h.

The *in vitro* bioactivity of the glasses was investigated using sintered glass pellets with similar dimensions and fabricated by a similar method: on the basis of DSC/HSM studies, the pellets were heated at 690 °C (B4) or 660 °C (B31 and B32) for 40 or 20 min (β = 20 °C/min), respectively, and subsequently sonicated in acetone for 5 min. The solubility and apatite forming ability of the pellets were investigated by soaking them in 30 mL of simulated body fluid (SBF) at 37 °C, pH = 7.4. The SBF solution had an ionic concentration (Na^+^ 142.0, K^+^ 5.0, Ca^2+^ 2.5, Mg^2+^ 1.5, Cl^−^ 148.8, HPO_4_^−^ 1.0, HCO_3_^2−^ 4.2, and SO_4_^2−^ 0.5 mmol·L^−1^) nearly equivalent to human plasma [29]. After filtering the SBF through sterilized filters (cameo 25 AS-MSI**,** pore size 0.22 µm), the glass-SBF mixtures were sealed into sterilized polyurethane flasks and placed in an incubator at 37 °C (±0.5 K) for 1, 3, 7, 14 and 21 days. The experiments were performed in triplicate to assure the accuracy of results. After each experiment, the glass pellets were removed from the solution, rinsed extensively with distilled water and dried at 100 °C for 1 h. The weight of the dried glass pellets, as well as the pH changes of the resulting SBF solutions were registered. Structural modifications occurring on the surface of glass samples were investigated by Fourier transform infrared spectroscopy (FTIR). For the measurements, 10 mg of the powders were loaded on the sample holder and the FTIR spectra acquired in a Cary 630 FT/IR (Agilent Technologies, Santa Clara, CA, USA) from 4000 to 400 cm^−1^ with 2 cm^−1^ resolution. The amorphous/crystalline nature of the glass surface, as well as qualitative analysis of crystalline phases, were determined by X-ray diffraction (XRD) analysis using a conventional Bragg-Brentano diffractometer (PANalytical, Almelo, The Netherlands) with Cu-Kα radiation. Data were recorded in the 2θ angle range between 3° and 90° (step size 0.02° and 19.7 s of counting time for each step) and the pattern analysis was carried out using X’Pert High Score software and the PCPDF data bank. Morphological and compositional modifications occurring on the surface of the pellets were investigated by scanning electron microscopy (SEM, Quanta Inspect 200, FEI, Hillsboro, OR, USA) with energy dispersive spectroscopy (EDS, model EDAX PV9900, EDAX Inc., Mahwah, NJ, USA). The analysis was done on Au sputtered samples and the pictures acquired at 15.0 kV.

## 4. Conclusions

The synthesis of new glasses with low Ca/P ratio and high bioactivity was successfully achieved. Furthermore, the synthesized glasses also presented a good thermal behavior, reflected by their high sintering ability. The synthesis of these new glass formulations may contribute to a change in the initial strategy for designing bioactive glasses, which has been extensively based on the theory that the highest bioactivity is achieved for glasses having low P_2_O_5_ (~6 wt %) amount in their composition. These results are expected to open new insights on the design of new glasses with enhanced properties for biomedical applications.

## Figures and Tables

**Table 1:** Formulations of the newly developed glasses.

Glass	Composition (wt %)							Ca/P Molar Ratio
	SiO_2_	P_2_O_5_	CaO	Na_2_O	K_2_O	MgO	CaF_2_	
B4	40.4	17.0	30.0	7.0	0.3	4.0	1.3	2.3
B31	40.0	14.0	30.0	10.5	0.5	3.0	2.0	2.8
B32	40.0	14.0	28.0	10.5	0.5	5.0	2.0	2.7

**Table 2:** Thermal parameters obtained from the DSC-TG/HSM analysis. *Tp*: Maximum crystallization temperature; STAB: Stability (*Tc*-*T_g_*); *T_g_*: Glass transition; *TFS*: Temperature first shrinkage; *TMS*: Temperature maximum shrinkage; Shrink, Shrinkage; *Tc*: onset of crystallization; *Sc*: Sintering ability (*Tc*-*TMS*). The temperature values are expressed in °C.

Glass	*Tp*1	*Tp*2	*Tp*3	*Tg*	*STAB*	*TFS*	*TMS*	*ΔT1*	Shrink1 (%)	Shrink until Tc (%)	Shrink2 (%)	Shrink Total (%)	*Tc*	*Sc*
B4	731	832	993	662	69	680	730	50	9.25	5.55	3.73	13.7	715	−15
B31	770	815	1020	623	117	650	730	80	16.55	16.55	2.76	19.31	740	10
B32	762	812	1036	620	107	640	730	90	18.06	18.06	1.42	19.48	727	−3

**Table 3:** Crystalline Phases of the synthesized bioglasses B4, B31 and B32. *Tp*: Crystallization peak.

Glass	*Tp*1	*Tp*2	*Tp*3
B4	Apatite	–	Apatite, Wollastonite, Diopside
B31	Apatite, Devitrite	Wollastonite, Apatite, Devitrite	Wollastonite, Apatite
B32	Devitrite, Apatite	Devitrite, Diopside, Apatite	Diopside, Wollastonite, Apatite
